# Emergency care capacity in Sierra Leone: A multicentre analysis

**DOI:** 10.1016/j.afjem.2024.01.003

**Published:** 2024-02-06

**Authors:** Zosia Bredow, Zoe Corbett, Moses Mohamed Tarawally, Lucy Jackson, Foday Tejan Mansaray, Santigie Sesay, Andrew Leather

**Affiliations:** a King's Global Health Partnerships, School of Life Course and Population Sciences, King's College London, London, UK; b Kenema Government Hospital, Kenema, Sierra Leone; c Connaught Hospital, Freetown, Sierra Leone; d Ministry of Health and Sanitation, Freetown, Sierra Leone

**Keywords:** Emergency care systems, Facility assessment, Service delivery, Toolkit

## Abstract

**Background:**

The Disease Control Priorities Project estimates that over 50 % of annual mortality in low- and middle-income countries can be addressed by improved emergency care. Sierra Leone's Ministry of Health and Sanitation has highlighted emergency care as a national priority. We conducted the first multicentre analysis of emergency care capacity in Sierra Leone, using the Hospital Emergency Unit Assessment Tool (HEAT) to analyse 14 government hospitals across the country.

**Methods:**

HEAT is a standardised assessment that is recommended in the World Health Organisation Emergency Care Toolkit. It has been used comparably elsewhere. To analyse Sierra Leone's emergency care capacity with the HEAT data, we created the HEAT-adjusted Emergency Care Capacity Score. Purposeful sampling was used to select 14 government facilities nationwide. A multidisciplinary team was interviewed over a 2-day in-person visit to each facility.

**Results:**

Human Resources was the strongest parameter, scoring 49 %. All hospitals provided emergency cover 24/7. Emergency Diagnostic Services was the most severely limited parameter, scoring 29 %. 3 hospitals had no access to basic radiography. Infrastructure scored 47 %. 2 hospitals had adequate electricity supply; 5 had adequate clean, running water. No hospitals had adequate oxygen supply. Clinical services scored 39 %. 10 hospitals had no designated Emergency Unit, only 2 triaged to stratify severity. Signal functions scored 38 %. No hospitals had reliable access to emergency drugs such as adrenaline. The total HEAT-adjusted Emergency Care Capacity Score across all hospitals was 40 %.

**Conclusions:**

These data identify gaps that have already led to local interventions, including focussing emergency resources to a resuscitation area, and training multidisciplinary teams in emergency care skills. This facility-level analysis could feed into wider assessment of Sierra Leone's emergency care systems at every level, which may help prioritise government strategy to target sustainable strengthening of national emergency care.

**African Relevance**

• Facility assessments for emergency care preparedness have been conducted in many countries across Africa; this is Sierra Leone's first beyond the capital city.
• Emergency Care Systems are limited across much of Africa; facility assessments are important sources of data for wider health system evaluation as found in the World Health Organisation's Emergency Care System Assessment.
• The Hospital Emergency Unit Assessment Tool (HEAT) has been developed to be context-appropriate for Emergency Unit evaluation in Africa; this is the first assessment using the tool in Sierra Leone.
• The Hospital Emergency Unit Assessment Tool-adjusted Emergency Care Capacity Score (HEAT-ECCS) indicates system gaps which may be used to guide and focus strategies to strengthen emergency care across other low-resource settings in Africa and elsewhere.

Alt-text: Unlabelled box

## Introduction

Effective emergency care is a vital component of population health, and ineffective emergency care systems contribute to preventable morbidity and mortality [1]. Illness or injury requiring emergency care accounts for 51 % of global mortality and 42 % of global burden of disease [2]. Despite this, emergency care for adults has not always been a global priority [3]. Facility assessments are important sources of data for wider health system evaluation and should be conducted alongside other methods to gain a full baseline assessment of the health system [4]. Done well, they can highlight service gaps and the resources required to improve care within health facilities. Facility assessments for emergency care preparedness have been conducted in many countries across Africa, including Namibia in 1998 [5], South Africa in 2008 [6], Morocco in 2011 [7], Kenya in 2012 [8], Tanzania in 2013 [9] and 2023 [10], Zambia in 2016 [11], Swaziland in 2017 [12] and the Kingdom of Eswatini in 2020 [13]. However, each of these assessments used different tools and methods, and as such the assessment results are difficult to directly compare. In response to this unmet need, the World Health Organisation (WHO) has developed an Emergency Care Toolkit to support the strengthening of adult emergency care systems [14]. WHO's systematic assessment tools include the Emergency Care Systems Assessment (ECSA) and the Hospital Emergency Unit Assessment Tool (HEAT) [14].

Sierra Leone, a West African country with a population of 8.4 million [15], is one of the least developed in the world, ranking 181 out of 189 countries on the United Nations’ Human Development Index [16]. Sierra Leone also has some of the world's worst health outcomes, due in part to deficiencies in health service delivery systems. Life expectancy at birth in 2021 was 60.1 years; the global life expectancy was 71.4 years [16]. A study published in 2022 found that 63 % of people die prematurely, and among young adults 47 % of non-maternity deaths are caused by malaria, other infectious diseases, injuries, or road traffic accidents, all of which could be addressed by high-quality emergency care [17].

Capacity scoring systems are a simple way to represent and compare data collected in facility assessments. Kushner et al. assessed surgical capacity in Sierra Leone in 2008 with the WHO's Tool for the Situational Analysis of Emergency and Essential Surgical Care (a surgical precursor to HEAT) [1,18]. This was reassessed by Groen et al. in 2011 with the Personnel, Infrastructure, Procedures, Equipment, and Supplies (PIPES) surgical tool [19]. Adult emergency care facilities have been evaluated in Sierra Leone's Capital by Coyle and Harrison in 2015 [20]. They reviewed 7 public and private hospitals in Freetown and found significant deficiencies across various indices, including human resources, training, drugs and equipment, and infrastructure [20]. Their study used an Emergency Care Capacity Score (ECCS), based on Baker et al.’s Emergency and Critical Care assessment (EaCC) in Tanzania [9]. However, there have been no studies to date of Sierra Leone's adult emergency care capacity outside of Freetown, where access to effective care is likely to be even more limited.

King's Sierra Leone Partnership (KSLP) has had a presence in Connaught Hospital, Freetown, since 2011. Researchers from KSLP, in partnership with the Ministry of Health and Sanitation (MOHS) and WHO, undertook this first country-wide multi-centre analysis of adult emergency care capacity across Sierra Leone using the WHO's HEAT and the HEAT-adjusted Emergency Care Capacity Score (HEAT-ECCS). We believe this score is universally applicable and useful for comparison with emergency care systems elsewhere.

## Methods

### The tool

HEAT is a standardised assessment that is recommended in the WHO's Emergency Care Toolkit [14]. It is included in Appendix A. HEAT assesses hospitals’ capacity to deliver effective emergency care, which, when used across multiple facilities, can inform a national emergency care system evaluation. The tool is generic, quick to complete, and appropriate for low resourced areas. The development of HEAT was informed by other tools including Emergency Obstetric Care assessment (EmOC), the African Federation for Emergency Medicine's Emergency Care Assessment Tool (ECAT) [21], the WHO Emergency Care System Framework [1], the WHO Guidelines for Essential Trauma Care [22], and the WHO Tool for the Situational Analysis of Emergency and Essential Surgical Care [23].

There are four question types:

A. Open-ended objective (e.g., name of facility)
B. Number response (e.g., number of emergency unit visits per year)
C. Discrete answers (e.g., yes or no)
D. HEAT Availability rating (1–3).

**Fig. 1:**
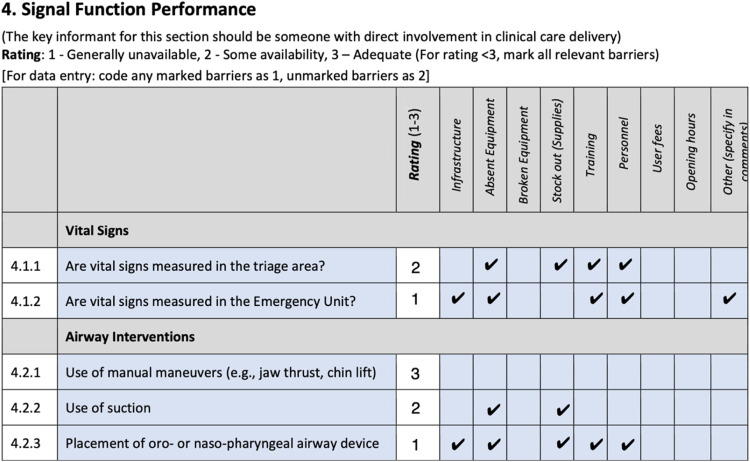
Example of barriers to availability coding in HEAT.

Each section includes areas for free-text to add depth to responses. Wherever an availability rating was answered with 1 or 2 (less than adequate), the barriers to availability were explored and coded to inform future study and change cycles [Fig. 1].

### Capacity score

The HEAT-ECCS is a composite score assessing the capacity of a system to provide emergency care. To create it, we mapped the WHO's HEAT questions to the 5 ECCS domains [Appendix A] [20]. The HEAT-ECCS can be calculated for an individual facility or calculated as an average of many facilities to indicate overall capacity within a region, or across an entire country as we have done. The HEAT-ECCS can also differentiate capacity scoring between the 5 domains. Due to its basis on a validated assessment in WHO's Emergency care Toolkit, the HEAT-ECCS is open access, reproducible, and standardised.

To generate the HEAT-ECCS, HEAT question types C (discreet answers) and D (availability ratings) are analysed. Open ended responses are qualitative and therefore not included in numerical analysis for scoring. To normalise data for analysis, all answers are mapped onto the availability score of 0–1 to keep data discrete [Table 1].

**Table 1:** HEAT answer types mapped to 0 – 1 availability score.

Discreet answers	Availability rating	Mapped availability score
1 - Yes	3 - adequate	1 - adequate
	2 - somewhat available	0.5 - somewhat available
2 - No	1 - generally unavailable	0 - generally unavailable

HEAT is categorised into 4 sections:

1. Facility Characteristics (including infrastructure and diagnostic services)
2. Human Resources
3. Clinical Services
4. Signal Function Performance

To map these sections to both Baker's and to Coyle's ECCS headings (Infrastructure; Diagnostics; Human resources; Systems; Guidelines; Training; Drugs and Equipment), the Facility Characteristics section of HEAT is subdivided into Infrastructure and Diagnostic Services [9,20]. Therefore the 5 sections for the HEAT-ECCS analysis are:

1. Infrastructure
2. Diagnostics Services
3. Human Resources
4. Clinical Services (incorporating systems and guidelines)
5. Signal Function Performance (incorporating training, drugs, and equipment)

Mean HEAT-ECCS scores are calculated for each section and a total score for each facility. Each section is weighted equally. Scores are expressed as percentages.

### Sampling

**Fig. 2:**
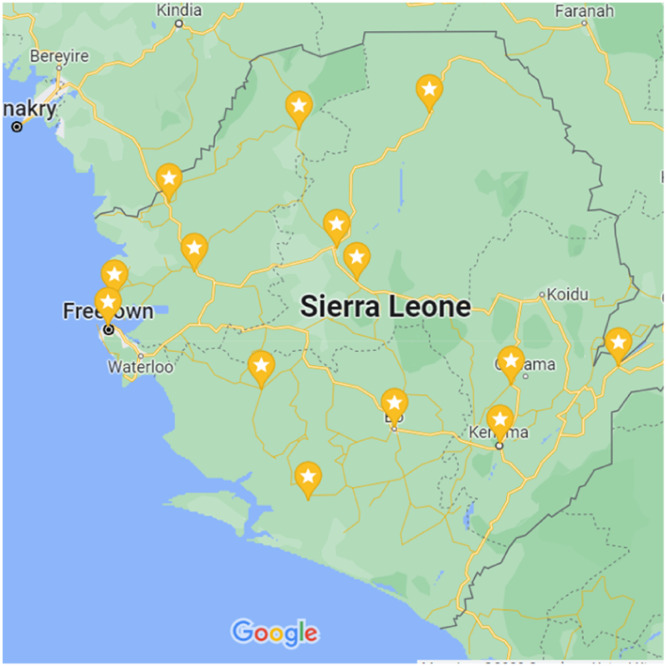
Map of Sierra Leone with locations of facilities sampled.

There are 16 districts in Sierra Leone which are subdivided into five provinces. Purposeful sampling was used to select 3 to 4 facilities in each of the 4 largest provinces (Northern, Eastern, Southern and North-Western) and 1 facility in the smallest province (Western Area). Facilities were selected by MOHS stakeholders and are the largest government facilities in each region, categorised by WHO ECSA as "First-level hospitals…the lowest level of hospital that receives referrals", thus capturing a stratification of adult emergency care capacity across Sierra Leone [14, Fig. 2].

Data collection took place between 4 November and 2 December 2021. The research team, from Sierra Leone and the UK, consisted of doctors, nurses, and a National Emergency Medical Services Referral Coordinator.

The first facility visit was conducted by the whole team together to standardise data collection methods and reduce investigator bias. Subsequent visits were made by binational teams of two researchers. Each visit lasted two days. The teams were re-randomised every two visits to maintain continuity of standards. Researchers conferred during visits, and all met virtually following each facility visit to ensure consensus between data within the standards set.

### Key informants (KI) selection

Medical superintendents of selected study facilities were informed by MOHS officials in writing and by the KSLP research team by telephone of the intention to visit and dates. Consent for data collection was obtained in person from the medical superintendent or their delegated deputy prior to commencement at each study facility. Researchers visited all relevant departments in person, and data were collected by speaking to workers (KIs) and looking at available resources. Verbal KI reports were found to differ from the team's in-person visit findings, therefore, to increase capacity score accuracy, availability was only rated as ‘adequate’ if the item was present and functioning (‘show us’ rather than ‘tell us’), and multiple KIs were involved per data section. KIs were selected based on their direct involvement in provision of emergency care; they are listed in Appendix C.

## Results

All raw data (numerical and open-ended word) were anonymised and entered into encrypted, password-protected files. No patient-identifying data were collected. Any sections with incomplete data were excluded from statistical analysis (18 data points out of 2436).

### HEAT – ECCS

**Fig. 3:**
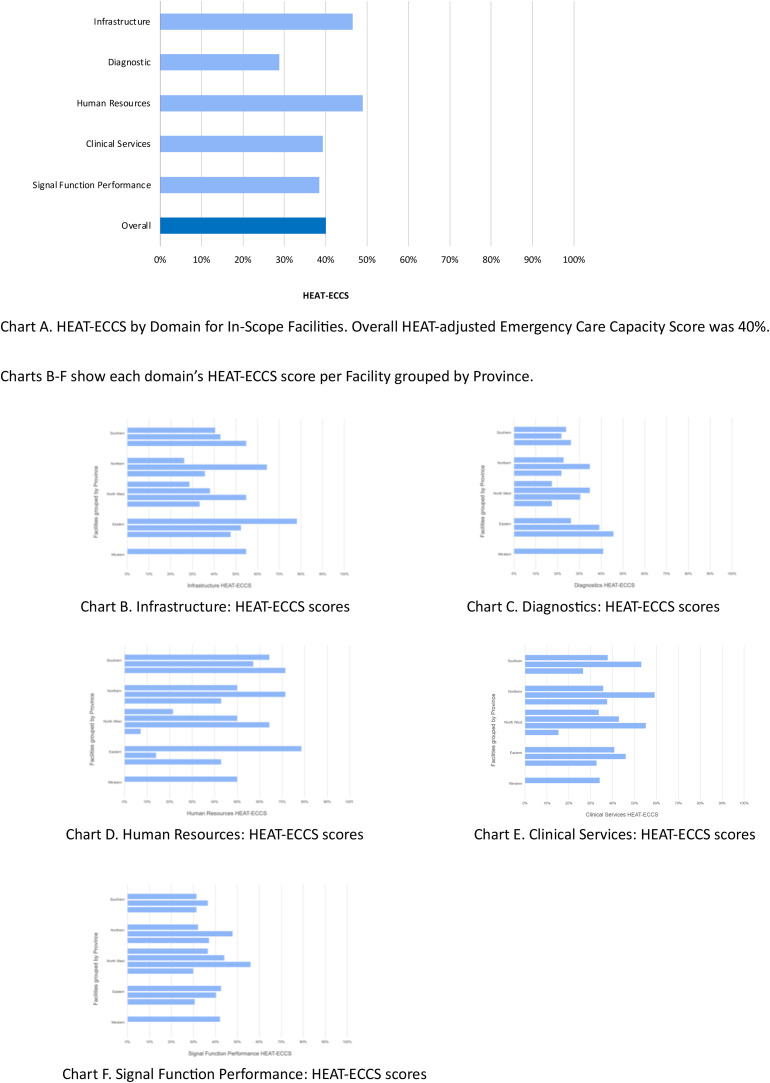
Charts to show HEAT-ECCS by Domain for In-Scope Facilities, collectively and per individual facility, grouped by Province.

The total HEAT-ECCS across all 14 facilities was 40 %. Capacity was highest in human resources and lowest in the sections of emergency diagnostic services and signal functions [Fig. 3]. No single facility was consistently strongest in all sections.

### Infrastructure

The HEAT-ECCS for Infrastructure across all facilities was 47 %. The range was 26–78 %.

9 facilities had no emergency or resus space. Instead, emergency patients and resources were throughout general wards. Only 2 facilities had adequate electricity supply, with 11 having ‘somewhat available’ electricity. As no facilities had piped oxygen, all relied on electricity-powered oxygen concentrators. Only 5 facilities had adequate clean, running water.

### Emergency diagnostic services

This was the most severely limited parameter; the HEAT-ECCS was 29 %. The range was 17–46 %.

3 facilities did not have X-ray facilities, and none scored X-ray as ‘adequate’. All facilities could test haemoglobin and 9 had laboratory access 24/7. Glucose test availability was only ‘adequate’ in 2 facilities. Many tests, including malaria and Human Immunodeficiency Virus (HIV) rapid diagnostic tests, were ‘generally unavailable’ due to absent equipment, reagent stock outs, training, user fees and limited lab opening.

Emergency diagnostic services in all facilities first had to be paid for by the patient or relatives (excepting <5 years or maternity patients under the Free Health Care Initiative) [24].

### Human resources

This was the strongest parameter with HEAT-ECCS of 49 %. The range was 7–79 %.

All facilities provided some emergency cover to wards 24/7. Nurses covered a 24/7 rota. Medical or community health officers covered on-calls, some off-site. Some care providers had a MOHS Personal Identification Number (PIN), so were salaried; other professionally qualified staff were unpaid workers awaiting PIN. On-call speciality services varied; for example, emergency surgery and anaesthesia were performed by medical superintendent and community health officer at one facility, and by consultant surgeon and anaesthetist at another.

Consulting services are shown in Table 2.

**Table 2:** Consulting Services Available to the Emergency Unit (EU) - or available to facilities’ emergency patients if no EU. Identified barriers to their availability were given as ‘Training’, ‘Personnel’ and ‘Opening Hours’.

	Number of facilities		
Service	Adequately available	Somewhat available	Generally unavailable
General Surgery	8	6	0
Obstetrics / Gynaecology	6	5	3
Orthopaedics	1	0	13
Anaesthesia	8	5	1
Paediatrics	7	2	5
Psychiatry	2	5	7
Other*	0	9	5

*Other Consulting Services at facilities: Eye; Dental; Palliative; HIV; Tuberculosis; Psychosocial; Special Care Baby Unit (SCBU); Malnutrition; Isolation; Family Planning; Genitourinary Medicine (GUM); Cervical Screening; Rehabilitation.

### Clinical services

The Clinical Services HEAT-ECCS was 39 %. The range was 15–59 %.

Only 2 facilities triaged to stratify severity and only 1 adequately measured vital signs in triage. No facility had clinical protocols (e.g., management of asthma, sepsis, etc.) or protocols for patient or staff safety, or emergency response.

### Signal functions

The Signal Functions HEAT-ECCS was 38 %; it is the most representative proxy for emergency clinical performance [21]. The range was 30–56 %. Signal function performance and barriers across facilities is shown in Appendix C.

4 facilities were adequately able to assess vital signs for emergency patients, although limited by lack of or broken equipment. 1 facility could adequately and 6 could somewhat adequately provide airway and breathing interventions (e.g., bronchodilators). For circulation, all facilities had at least some availability for peripheral intravenous access. For sepsis, all facilities had at least some availability to administer intravenous antibiotics. For trauma, all facilities had at least some availability to perform initial wound care. No facilities had adequate reliable or free access to adrenaline.

## Discussion

In this multicentre analysis of emergency care capacity in Sierra Leone, we found that all assessed facilities provided some level of emergency care, but that there were severe limitations to capacity, notably in the domains of emergency diagnostic services, infrastructure, clinical services, and signal functions, as reflected in the HEAT-ECCS.

This is the first study to create a capacity scoring system based on HEAT, distilling the 223 HEAT data points to produce a single meaningful, reproducible score. The HEAT-ECCS enables direct comparison of emergency care capacity regionally, nationally, and internationally, and provides a measure for change if data collection is repeated.

This is the first study to describe the capacity of adult emergency care systems across Sierra Leone. Previous studies reviewing the country's emergency care have focussed on the Capital, Freetown. The 7 public and private hospitals in Freetown assessed in Coyle's ECCS averaged a score of 60 % [20]. The HEAT-ECCS across all 14 facilities in our study was 40 %. This suggests that the government facilities that we assessed in the rural districts were less well prepared for emergency patients than in the Capital, which is supported by our data.

An assessment of surgical capabilities in 10 Sierra Leonean hospitals, including 5 outside Freetown, was published in 2008 by Kushner et al. using the WHO's Tool for the Situational Analysis of Emergency and Essential Surgical Care [18], and then again in 2011 by Groen et al. using the PIPES tool [19]. The 5 facilities outside Freetown were all included in our assessments as well as in both Kushner's and Groen's. The data for these facilities in both Kusher's and Groen's studies is comparable to our results, particularly in the domains of infrastructure and equipment. As in our study, Groen's study noted both that the “capacity within Freetown was much greater than in the district hospitals” and that since Kushner's 2008 assessment “there were still great deficiencies in basic infrastructure elements, such as running water or electricity” [19].

HEAT has been used in a comparative study assessing 3 referral hospitals in Eswatini [13], and more recently, assessing 11 facilities in Tanzania [10]. The diagnostic services findings are similar between the 3 studies, although there was higher availability across signal function performance in the Eswatini and Tanzania facilities compared with Sierra Leone [10,13].

It is notable that our data show wide ranges of HEAT-ECCS both within and between facilities. We did not find strong correlation between any single factor and overall HEAT-ECCS, which suggests that there is no golden key when strengthening a system's emergency care capacity. Our source data show weak correlation between oxygen availability ratings and HEAT-ECCS in sections of emergency diagnostic services, infrastructure, and signal functions. There was positive correlation between availability of electricity and signal functions HEAT-ECCS. We suggest that local and national resource prioritisation to these facilities will therefore influence their emergency care capacity in these domains.

Between facilities we witnessed many approaches to addressing the same challenges. Interestingly, successes and weaknesses were not often shared regionally, or nationally. This may have been due to facilities working in silos, which may reflect the wide HEAT-ECCS ranges in our data. During the project we supported MOHS employees to create and participate in regional communities of practice via WhatsApp, to facilitate sharing of best practices and inter-facility problem solving.

This study has certain limitations. Firstly, as it was not possible for our team to survey all facilities in Sierra Leone, no facilities funded privately or by non-governmental organisations were included. By omitting them we have not included every district in Sierra Leone. Second, as this study focussed specifically on adult emergency care, we omitted the HEAT data on paediatric and obstetric interventions. As these interventions fall under the funding prioritisation of Sierra Leone's Free Health Care Initiative it would be interesting to see if there is capacity discrepancy between these and the adult emergency care that we have assessed. This requires further study. Third, all the sections in the HEAT-ECCS were taken directly from the HEAT form. Data collection was therefore limited by the knowledge of those KIs answering HEAT questions, and the scope of the tool itself. For example, there is no specific HEAT question to capture the number of salaried vs unpaid members of staff, and as such any data was recorded as qualitative comments and not included in numerical analysis. Our team mitigated KI limitations by undertaking in-person data collection, and by the breadth and number of KIs interviewed at each site. Finally, not all adult emergency data collected in the HEAT was analysed for this study as we did not include question types A and B for the HEAT-ECCS analysis. For example, the Human Resources analysis was based on the consulting services available to the EU, and not on the other HEAT questions in this section, as these were total number responses (question type B) such as ‘number of non-rotating and rotating providers assigned to EU’. Although disseminated to each facility, we did not analyse the qualitative data for this study.

## Conclusions

These data and the HEAT-ECCS provide evidence of emergency care capacity across 14 of Sierra Leone's district and regional hospitals, broadening the contextual understanding of the facilities in which systems change is being delivered. We believe that creating a simple capacity score that is universally applicable and based on WHO's Emergency Care Toolkit allows for clear comparison between regions within a single country or globally. Our aim is to create practical outputs for policymakers and future researchers. Sharing our findings with the facilities assessed has already led to some local interventions, including implementing triage systems, focussing emergency resources to a single resuscitation area, and training multidisciplinary teams in emergency care skills. This facility-level analysis could feed into wider assessment of Sierra Leone's emergency care systems at every level, which may then assist MOHS prioritisation of gaps to target as part of an ECSA prioritisation process. The approach described here could be used to guide and focus national strategies to strengthen emergency care across Sierra Leone and other low-resource settings.

## Dissemination of results

Results from this study were shared with the key informants, emergency care staff and stakeholders at each of the facilities visited, both in person at round table multidisciplinary team discussion and by written facility reports that suggested strengths and areas for potential improvement. Facilities were encouraged to share both strengths and suggested areas for improvement within their regional community of practice WhatsApp groups to promote inter-facility problem solving. Study data and analysis was also shared with emergency care stakeholders in Sierra Leone's MOHS and WHO. There are current plans in place for a national re-evaluation with aims to assess capacity in all government facilities in Sierra Leone as part of the MOHS Emergency Care Systems Assessment, supported by WHO. The initial prioritisation exercise started in 2022, and the re-evaluation hopes to extend to collect data on Sierra Leone's Emergency, Critical and Operative care capacity as part of the newly created WHO Emergency Care Toolkit.

## Authors’ contribution

Authors contributed as follows to the conception or design of the work; the acquisition, analysis, or interpretation of data for the work; and drafting the work or revising it critically for important intellectual content:

*ZB contributed 55 %; ZC 15 %; AL 10 % and MMT, LJ, FTM, and SS contributed 5 % each.*

All authors approved the version to be published and agreed to be accountable for all aspects of the work.

## Declaration of competing interest

The authors declared no conflict of interest.

## Appendix Supplementary materials

Appendix A. World Health Organisation Hospital Emergency Unit Assessment Tool (HEAT).

Appendix B. List of Key Informants by role; facility units Key Informants cover.

Appendix C. Signal function performance, availability count and identified barriers to availability (frequency across facilities) for all HEAT signal functions.
