# Supplementary material for: Emergency care capacity in Sierra Leone: A multicentre analysis
Source: Afr J Emerg Med. 2024 Feb 6;14(1):58–64. doi: 10.1016/j.afjem.2024.01.003 (PMC10859259; doi:10.1016/j.afjem.2024.01.003)
Supplement: Supplementary file 1 [file mmc1.pdf]

Appendix B.

World Health Organisation Hospital Emergency Unit Assessment Tool (HEAT).

WHO
EMERGENCY UNIT
ASSESSMENT TOOL

World Health Organization

DRAFT 6 November 2017

For more information, contact: reynoldst@who.int

Table of Contents

Introduction

- How to use this tool
- Scoring system
- Signal Functions

Emergency Unit Assessment

1. Facility Characteristics
   - 1.1 Identifying Information
   - 1.2 Facility Metrics
   - 1.3 Infrastructure and essential equipment
   - 1.4 Diagnostic Services
2. Human Resources
   - 2.1 Emergency Care Clinical Providers
   - 2.2 Consulting services Available to the Emergency Unit
3. Clinical Services
   - 3.1 Access
   - 3.2 Triage
   - 3.3 Guidelines, protocols and checklists
   - 3.4 Ancillary Services
   - 3.5 Quality improvement
4. Signal functions
   - 4.1 Vital Signs
   - 4.2 Airway Interventions
   - 4.3 Breathing Interventions
   - 4.4 Cardiac Interventions
   - 4.5 Neurologic Interventions
   - 4.6 Sepsis Interventions
   - 4.7 Trauma Interventions
   - 4.8 Obstetric Interventions
5. Essential Resources for Emergency Care

Introduction

Emergency care addresses a wide-range of medical, surgical, and obstetric conditions, including injury, complications of pregnancy, exacerbations of non-communicable diseases (e.g. heart attacks, strokes), and acute infections (e.g. sepsis, malaria). Particularly in areas where barriers to care exist, emergency units are often the first point of contact with the healthcare system. Emergency care is a critical component of universal health care, and with sound planning and organization, has the potential to address conditions causing over half of deaths and a third of disability incurred annually in low- and middle-income countries.

A strategic assessment of emergency care capacity at healthcare facilities is among the first steps in the planning process. Findings can be used to identify gaps and target interventions at both individual facilities and across the healthcare system more broadly; in addition, periodic assessments may also be useful for monitoring capacity over time.

This assessment tool designed to evaluate the structure and key functions of an emergency unit (or any dedicated intake area for acutely ill and injured patients). It is derived from the WHO *Emergency Care System Framework*, WHO *Guidelines for Essential Trauma Care*, WHO *Tool for Situational Analysis to Assess Emergency and Essential Surgical Care* and the African Federation for Emergency Medicine *Emergency Care Assessment Tool*, and was informed by a broad review of other relevant instruments.

How to use this tool

This tool is designed to assess emergency care capacity and organization at the facility level. It can be used at an individual facility or across a group of facilities region- or country-wide. Hospital administrators and health system planners can use the findings to identify gaps in order to guide planning. If the tool is to be used at the regional or country level, either all facilities within a certain category should be assessed (all regional or district hospitals, for example), or a robust sampling strategy should be utilized to ensure that the facilities assessed best represent the regional or national reality. Sampling strategies for facility-based assessments include:

- Exhaustive sampling –all facilities in the target area (e.g., region, country) or of a specific type are assessed (eg. all district hospitals).
- Random sampling (including stratified and cluster random sampling) – facilities to be assessed are randomly selected from among a target group
- Purposeful sampling – facilities are chosen by well-informed stakeholders specifically to reflect the diversity of facilities, geography, administrative areas, patient volume, levels of care, etc.;

Exhaustive sampling of all relevant facilities is the most robust approach, but may not always be feasible. Considerations for choosing a sampling strategy include: objectives of the assessment, needs of stakeholders, total number and types of facilities, heterogeneity of the country or region (differences between urban and rural areas, for example), and need for statistical certainty. For more details on sampling strategies and sample size calculations for facility-based capacity assessments, see the WHO *Service Availability and Readiness Assessment* (SARA): http://www.who.int/healthinfo/systems/SARA_Implementation_Guide_Chapter2.pdf.

Prior to performing the assessment, permission should be sought and granted by the appropriate national agencies and facility administrators. The facility administration can also help identify key informants to participate in the assessment. Ideally, more than one key informant is approached to complete the tool to allow triangulation of responses, which may improve the accuracy of the results. If more than one key informant completes all or the respective part of the tool, there are multiple ways to compile the results: i) the median response for each question becomes the final response; or ii) the response of the key informant with the most understanding of a specific resource, service or function becomes the final response; or iii) a consensus-process is initiated (eg, a meeting convened) and a single answer for each question agreed upon by respondents.

Potential key informants that might provide important information for this assessment include:

- Facility administrators (e.g., medical director, human resources director, operations officer, nurse matron);
- Providers (e.g., nurses, clinical officers, specialists) who work in the emergency unit;
- Laboratory and radiology unit technicians;
- Technicians and biomedical engineers who interact with equipment in the aforementioned units/departments;
- Procurement and medical stores staff;
- Facility statistics and health information staff.

Note that not all facilities will have staff in all of these positions, or even have each of the units/departments above. In addition, the informant with the highest authority at the facility may not be the informant with the most accurate understanding of the availability of a given resource. For the section on signal functions, the key informant should someone with direct involvement in clinical care delivery.

It should also be noted that this tool does not define a minimum standard for every emergency unit at every level of the health system. It is intended as a general tool to identify gaps that can be addressed by implementation of standards promoted elsewhere. Ultimately, countries will need to determine which services they aim to provide at a given level of the health system.

Scoring system

There are four question types in this assessment:

1. Open-ended (e.g., name of facility);
2. Number response (e.g., number of emergency unit visits per year);
3. Discrete answers (e.g., yes or no);
4. Availability rating.

The availability rating questions are used to assess resource and service capacities, specifically the ability to perform key functions in the time frame needed for emergency care. These questions are meant to reflect the demand-side factors (e.g., number of patients in need) for the service, as well as the supply-side factors (e.g., sufficient resources, satisfactory training). For each of these questions, the resource or service should be noted as:

1 - Generally unavailable;
2 - Somewhat available (available to **ONLY SOME** of those who need it);
3 - Adequate (**PRESENT and AVAILABLE** to almost everyone in need, and used when needed).

If the availability rating is less than 3 (less than adequate), it is important to know the factors that contribute to its deficiency. Common factors that contribute to inadequate resources (such as supply chain problems, or lack of training) can then be identified and addressed. Therefore, for ratings less than 3, the person administering the survey should systematically prompt the key informant to identify reasons for the less than adequate rating; more than one factor can be marked per resource, service or function.

- *Infrastructure* - physical space, electricity, water
- *Absent equipment* – the resource is not present at the facility
- *Broken equipment* –the resource is present, but not in working order
- *Stock out* – the resource or function cannot be procured, or required supplies out of stock often due to stock management practices or procurement failures (e.g., reagents, tubes, IV catheters)
- *Training* – staff knowledge/skill gaps limit capacity to use the resource or perform a function

- *Personnel* - resource, service or function available, but lack of adequate numbers of staff limit capacity
- *User fees* - resource or function available, but out-of-pocket payment requirement prevents care delivery
- *Opening hours* - hours the facility can be accessed by acute patients
- *Other* – enter explanation in comments field

Signal Function Performance

Emergency care is a cross-cutting, service delivery innovation providing timely intervention for acute conditions such as sepsis or trauma. The capacity to perform key time-dependent interventions for these sentinel conditions can be used as a marker of overall emergency unit performance. Any limited availability of these key interventions signals a critical gap in emergency care delivery capacity. Use of these “signal functions” allows for a rapid, simple assessment and the identification of failures of emergency care delivery whose cause can then be identified and addressed. For example, identifying limited availability of intravenous volume resuscitation should prompt evaluation for causes such as lack of functioning equipment, supply stock-outs, gaps in staff skills/knowledge, or poor infrastructure.

Emergency Care Delivery Assessment

1. Facility Characteristics

1.1 Identifying Information

1.1.1	**Date**	
1.1.2	**Country**	
1.1.3	**Name of facility**	
1.1.4	**Address of facility** (include city, state or province)	
1.1.5	**GPS Reading** (if available)	Degrees Minutes Seconds<br>Latitude:<br>Longitude:
1.1.6	**Name** person filing out form	
1.1.7	**Facility Contact(s)**	**1. Name:** **Phone:** **Email:**<br>**2. Name:** **Phone:** **Email:**
1.1.8	**Level of facility***	□ Health centre or clinic(1) □ 1st level hospital(2) □ 2nd level hospital(3) □ Tertiary hospital(4)
1.1.9	**Type of facility**	□ Private hospital(1) □ NGO hospital(2) □ Government hospital(3)
1.1.10	Distance to nearest higher level facility:	
1.1.11	Is there an area (room, unit, department) specifically designated for emergency care? **Yes(1)** **No (2)**	
1.1.12	Population served by facility (e.g., 123,000):	
1.1.13	Interview Start Time (Use 24 hr clock system):	

*Footnote: see reference definitions

1.2 Facility Metrics

	Descriptor	Number
1.2.1	Emergency unit visits per year	
1.2.2	Outpatient visits per year (excluding emergency unit visits)	
1.2.3	Inpatient admissions per year	
1.2.4	Beds/gurneys dedicated for general emergency care (not including inpatient beds)	
1.2.5	Inpatient hospital beds	
1.2.6	Functioning operating theatres (24/7)	
1.2.7	Functioning high acuity unit (e.g. ICU) beds with capacity for continuous monitoring and mechanical ventilation	
1.2.8	Emergency operations per year	

	**Available hours**	
1.2.9	During which hours is the emergency unit covered by providers who are physically present in the unit?	
1.2.10	During which hours is the emergency unit covered by providers who are on call, inside the facility?	
1.2.11	During which hours is the emergency unit covered by providers who are on call outside the facility?	
	Opening hours of:	
1.2.12	Emergency Unit	
1.2.13	Laboratory	
1.2.14	Pharmacy	
1.2.15	Radiology	
1.2.16	Operating Theater	
1.2.17	**Comments:**	

1.3 Infrastructure and essential equipment

Rating: 1 - Generally unavailable, 2 - Some availability, 3 – Adequate

	**Infrastructure Element**	**Rating** (1-3)	**Comments** (if rating <3)
1.3.1	Clean, running water		
1.3.2	Electricity source (e.g., wired, generator)		
1.3.3	Designated telephone or radio for communicating with other facilities and/or prehospital providers		
1.3.4	Paper-based emergency unit chart		
1.3.5	Electronic emergency unit chart		
1.3.6	Isolation room for infectious diseases (e.g., TB, haemorrhagic fever)		
1.3.7	Easy physical access to emergency unit for those requiring a wheelchair or stretcher		
1.3.8	Designated waiting area		
1.3.9	Designated triage area		
1.3.10	Designated resuscitation area		
1.3.11	Personal protective equipment (e.g., hair covers, eye protection, N95 face masks, impermeable gowns, shoe covers, gloves) in a range of sizes		
1.3.12	Electronic cardiac monitoring in emergency unit		
1.3.13	Crash trolley or code cart with high-acuity equipment and supplies of various sizes in emergency unit		
1.3.14	Rapid access to a transport ambulance and provider to administer care during transport for patients who need to be transferred to another facility		
1.3.15	Is there a dedicated mechanism (radio, telephone) for communication with other facilities for transfer of patients?		
1.3.16	Is there access to storage space within (or with immediate proximity to) the emergency unit, including secure storage for controlled substances?		
1.3.17	Access to dedicated staff work area		
1.3.18	Access to toilet facilities for patients and staff		
1.3.19	Access to handwashing facilities in each patient care area		
1.3.20	System for stocking, managing, and dispensing medications in emergency unit		
1.3.21	Oxygen in emergency unit		

	Which of the following methods supply oxygen in this unit?:	Yes	No
1.3.22	Oxygen is supplied through a central piped system	1	2
1.3.23	Oxygen is supplied by oxygen concentrator stored on this unit	1	2
1.3.24	Oxygen is supplied in tanks that are stored on this unit	1	2
1.3.25	Emergency unit calls for tank of oxygen from central location if needed	1	2
1.3.26	Emergency unit calls for oxygen concentrator from central location if needed	1	2
1.3.27	**Comments:**		

1.4 Diagnostic Services

Rating: 1 - Generally unavailable, 2 - Some availability, 3 – Adequate (For rating <3, mark all relevant barriers)
[For data entry: code any marked barriers as 1, unmarked barriers as 2]

	Descriptor	***Rating (1-3)***	*Infrastructure*	*Absent Equipment*	*Broken Equipment*	*Stock out (Supplies)*	*Training*	*Personnel*	*User fees*	*Opening hours*	*Other (specify in comments)*
	**Laboratory-based Testing**										
1.4.1	Hemoglobin										
1.4.2	Full blood count										
1.4.3	Coagulation profile (PT/PTT)										
1.4.4	Electrolytes										
1.4.5	BUN and creatinine										
1.4.6	Lipase										
1.4.7	Cardiac marker (e.g., troponin)										
1.4.8	Arterial blood gas										
1.4.9	Cross matching for blood and blood products										
1.4.10	Blood cultures										
1.4.11	Capacity to obtain sterile blood samples for lab testing										
1.4.12	System for reporting lab results in a timely fashion										
	**Point of Care Testing – available in the emergency unit**										
1.4.13	Urine dipstick										
1.4.14	Urine pregnancy										
1.4.15	Glucose										
1.4.16	Malaria Rapid Diagnostic Test (RDT)										
1.4.17	Rapid HIV testing										
	**Diagnostic imaging**										
1.4.18	Stationary X-ray										
1.4.19	Portable X-ray for use in emergency unit										
1.4.20	Ultrasound in the hospital										
1.4.21	Ultrasound for use in emergency unit										
1.4.22	CT scan										
1.4.23	System for reporting radiology results in a timely fashion										
1.4.24	**Comments:**										

2. Human Resources

2.1 Emergency Care Clinical Providers

2.1.1	Do you have a core of fixed (non-rotating) providers permanently assigned to the emergency unit?	Yes (1)	No (2)

	Descriptor	Total Number	Number of licensed or certified
	**Number of <u>non-rotating</u> providers assigned to emergency unit**		
2.1.2	Nurses		
2.1.3	Mid-level provider or advance practice nurses (e.g., clinical officers)		
2.1.4	Medical officers (doctors without specialist training)		
2.1.5	Emergency medicine specialists		
2.1.6	Other specialist doctor		
	**Number of <u>rotating</u> providers assigned to emergency unit**		
2.1.7	Nurses		
2.1.8	Mid-level provider or advance practice nurses (e.g., clinical officers)		
2.1.9	Medical officers (e.g., doctors without specialist training)		
2.1.10	Emergency medicine specialists		
2.1.11	Other specialist doctor		
2.1.12	**Comments:**		

2.2 Consulting Services Available to the Emergency Unit

Rating: 1 - Generally unavailable, 2 - Some availability, 3 – Always available

	Consulting Service	Rating (1-3)	Comments
2.2.1	General Surgery		
2.2.2	OB/GYN		
2.2.3	Orthopedics		
2.2.4	Anesthesia		
2.2.5	Paediatrics		
2.2.6	Psychiatry		
2.2.7	Other (Please list):		

3. Clinical Services

3.1 Access

3.1.1	What proportion of patients with emergency conditions are brought to the facility by ambulance with formally trained prehospital care providers?	_____ %	Don't know
3.1.2	Are there regulations and/or protocols mandating that acutely ill or injured patients are clinically triaged prior to being required to register?	Yes(1)	No(2)
3.1.3	Does the facility require payment prior to provision of initial emergency care?	Yes(1)	No(2)
3.1.4	Is there an electronic system for registration?	Yes(1)	No(2)
3.1.5	**Comments:**		

3.2 Triage

		Yes	No
3.2.1	Does this facility use a formal triage system (includes a structured triage tool used by trained personnel 24 hours per day, 7 days per week)?	1	2
3.2.2	Are there time targets for each triage category (e.g., YELLOW – seen by provider within 2 hours)?	1	2
3.2.3	If there are time targets, is compliance tracked regularly?	1	2
3.2.4	Are there specific triage protocols for children <5 years of age?	1	2
3.2.5	Are there specific triage protocols for pregnant women?	1	2
3.2.6	Are vital signs measured in triage area?	1	2
3.2.7	**Comments:**		

3.3 Guidelines, protocols and checklists

	Are the following protocols available at this facility?	Yes	No
3.3.1	Protocol for systematic triage that ensures patients are seen in order of acuity	1	2
3.3.2	Syndromic surveillance guidelines with links to public health officials for case definition and reporting	1	2
3.3.3	Clear protocol for communication with hospital administration during times of overcrowding	1	2
3.3.4	Emergency unit specific emergency response protocol, including protocol for mass casualty incidents	1	2
	**Are the following clinical management protocols available at this facility?**		
3.3.5	Protocol for initial approach to ABCs (airway, breathing, circulation, etc.) and basic neurologic function	1	2
3.3.6	Trauma care checklist	1	2
3.3.7	Medical resuscitation checklist	1	2
3.3.8	Protocol for neonatal resuscitation	1	2
3.3.9	Protocol for volume resuscitation of children and adults	1	2
3.3.10	Protocol for adjusting interventions for malnourished patients	1	2
3.3.11	Protocol for post-exposure prevention of STI/HIV, emergency contraception, counseling	1	2
3.3.12	Protocol for management of labor and delivery in low risk women	1	2
	Condition-specific management protocols for:		
3.3.13	Asthma exacerbation	1	2
3.3.14	Pneumonia	1	2
3.3.15	Maternal hemorrhage	1	2
3.3.16	Sepsis	1	2
3.3.17	Diabetic ketoacidosis	1	2
3.3.18	Other: _________________________	1	2
	**Are the following disposition protocols available at this facility?**		
3.3.19	Acuity-based internal transfer protocols to OR or ICU	1	2
3.3.20	Protocol for timely disposition from the emergency unit	1	2
3.3.21	Protocol for conveying information about discharge or disposition to the patient	1	2
3.3.22	Hand-over protocols when transferring patients from one care provider to another	1	2
	**Are the following outside transfer protocols available at this facility?**		
3.3.23	Condition-specific transfer or referral protocols (e.g., criteria for transfer of burn patient to burn centre)	1	2
3.3.24	Communication with receiving facility prior to transfer of patients with emergency conditions	1	2

	Are the following safety protocols available at this facility?	Yes	No
3.3.25	Infection prevention and control protocols	1	2
3.3.26	Protocol for post exposure prophylaxis for health care workers	1	2
3.3.27	Security protocols to protect staff, patients, and infrastructure from violence.	1	2
3.3.28	Protocol for managing hazardous exposures (including designated decontamination area)	1	2
3.3.29	Containment and disposal of sharps and biomedical waste	1	2
3.3.30	Plan to ensure emergency unit staff and patient safety if an incident occurs *within* the emergency unit (including space, transport, communications)	1	2
3.3.31	**Comments:**		

3.4 Ancillary Services

Rating: 1 - Generally unavailable, 2 - Some availability, 3 – Adequate (For rating <3, mark all relevant barriers)
[For data entry: code any marked barriers as 1, unmarked barriers as 2]

	Descriptor	***Rating (1-3)***	*Infrastructure*	*Absent Equipment*	*Broken Equipment*	*Stock out (Supplies)*	*Training*	*Personnel*	*User fees*	*Opening hours*	*Other (specify in comments)*
3.4.1	Social work services										
3.4.2	Patient transport services (within emergency unit)										
3.4.3	Security										
3.4.4	**Comments:**										

3.5 Quality improvement

		Yes	No
3.5.1	Systematic process for collecting patient data that links condition, management and outcomes (e.g., trauma registry)	1	2
3.5.2	Regular meetings convened to use clinical data for quality improvement (e.g., morbidity and mortality conferences, preventable death panels)	1	2
3.5.3	Tracking (e.g., clinical audit) to ensure that quality improvement actions (e.g., corrective action) are implemented after review meetings	1	2
3.5.4	Clinical document template (e.g., standardized clinical chart)	1	2
3.5.5	Has there been a visit to this emergency facility by a supervisor from outside the facility within the last 6 months?	1	2
3.4.6	Is there any documentation from the most recent external supervisory visit?	1	2
3.4.7	Does the document provide any feedback or comments on some aspect of emergency services?	1	2
3.4.8	**Comments:**		

4. Signal Function Performance

(The key informant for this section should be someone with direct involvement in clinical care delivery)
Rating: 1 - Generally unavailable, 2 - Some availability, 3 – Adequate (For rating <3, mark all relevant barriers)
[For data entry: code any marked barriers as 1, unmarked barriers as 2]

		Rating (1-3)	*Infrastructure*	*Absent Equipment*	*Broken Equipment*	*Stock out (Supplies)*	*Training*	*Personnel*	*User fees*	*Opening hours*	*Other (specify in comments)*
	**Vital Signs**										
4.1.1	Are vital signs measured in the triage area?										
4.1.2	Are vital signs measured in the Emergency Unit?										
	**Airway Interventions**										
4.2.1	Use of manual maneuvers (e.g., jaw thrust, chin lift)										
4.2.2	Use of suction										
4.2.3	Placement of oro- or naso-pharyngeal airway device										
4.2.4	Placement of supraglottic device										
4.2.5	Endotracheal intubation										
4.2.6	Creation of surgical airway										
	**Breathing Interventions**										
4.3.1	Measurement of pulse oximetry at triage										
4.3.2	Measurement of pulse oximetry in emergency unit										
4.3.3	Administration of critical therapies for reactive airway disease										
4.3.4	Administration of oxygen										
4.3.5	Bag-valve-mask ventilation										
4.3.6	Non-invasive mechanical ventilation										
4.3.7	Invasive mechanical ventilation										
4.3.8	Perform needle decompression of tension pneumothorax										
4.3.9	Placement of chest tube										
4.3.10	**Comments:**										

Rating: 1 - Generally unavailable, 2 - Some availability, 3 – Adequate (For rating <3, mark all relevant barriers)
[For data entry: code any marked barriers as 1, unmarked barriers as 2]

	CIRCULATION	***Rating*** (1-3)	*Infrastructure*	*Absent Equipment*	*Broken Equipment*	*Stock out (Supplies)*	*Training*	*Personnel*	*User fees*	*Opening hours*	*Other (specify in*
	**Volume Resuscitation Interventions**										
4.4.1	Administer oral rehydration										
4.4.2	Adjust fluid resuscitation for malnutrition or severe anaemia										
4.4.3	Place peripheral IV access										
4.4.4	Establish intraosseous access										
4.4.5	Perform venous cutdown										
4.4.6	Establish central venous access										
4.4.7	Administration of IV fluids										
4.4.8	Place urinary catheter										
	**Control of Bleeding**										
4.5.1	External control of haemorrhage										
4.5.2	Perform packing and/or suture control										
4.5.3	Apply arterial tourniquet										
4.5.4	Apply pelvic binding or sheeting										
4.5.5	Ability to perform safe transfusion (including protocols for appropriate ratios for massive transfusion)										
4.5.6	Perform and interpret point of care ultrasound										
	**Cardiac Interventions**										
4.6.1	Perform and interpret ECG										
4.6.2	Administer aspirin for ischemia										
4.6.3	Perform external defibrillation and/or cardioversion										
4.6.4	Perform external cardiac pacing										
4.6.5	Administration of adrenaline										
4.6.6	Administration of thrombolytics										
4.6.7	Perform pericardiocentesis										
	**Comments:**										

Rating: 1 - Generally unavailable, 2 - Some availability, 3 – Adequate (For rating <3, mark all relevant barriers)
[For data entry: code any marked barriers as 1, unmarked barriers as 2]

	**Neurologic Interventions**	***Rating*** (1-3)	*Infrastructure*	*Absent Equipment*	*Broken Equipment*	*Stock out (Supplies)*	*Training*	*Personnel*	*User fees*	*Opening hours*	*Other (specify in comments)*
	**Unconscious patient**										
4.7.1	Protect from secondary injury										
4.7.2	Check and/or administer glucose										
4.7.3	Administer insulin for hyperglycemia										
4.7.4	Perform lumbar puncture										
	**Seizure**										
4.7.5	Administer benzodiazepine										
4.7.6	Administer IV magnesium for pregnant patient										
4.7.7	Administer locally appropriate antidote										
	**Other**										
4.7.8	Perform mental status examination										
4.7.9	Management of extreme temperatures										
4.7.10	Administer appropriate therapeutics for agitation										
4.7.11	Ability to provide physical restraints										
4.7.12	Perform procedural sedation										
	**Comments:**										

	**Sepsis Interventions**	***Rating*** (1-3)	*Infrastructure*	*Absent Equipment*	*Broken Equipment*	*Stock out (Supplies)*	*Training*	*Personnel*	*User fees*	*Opening hours*	*Other (specify in comments)*
4.8.1	Administration of IV antibiotics										
4.8.2	Administration of IV vasopressors										
4.8.3	Perform diagnostic paracentesis										
4.8.4	Bedside minor surgical techniques for source control (e.g., abscess, empyema)										
	**Comments:**										

Rating: 1 - Generally unavailable, 2 - Some availability, 3 – Adequate (For rating <3, mark all relevant barriers)
[For data entry: code any marked barriers as 1, unmarked barriers as 2]

	**Trauma Interventions**	***Rating*** (1-3)	*Infrastructure*	*Absent Equipment*	*Broken Equipment*	*Stock out (Supplies)*	*Training*	*Personnel*	*User fees*	*Opening hours*	*Other (specify in comments)*
4.9.1	Perform initial appropriate wound care										
4.9.2	Administer tetanus vaccination or IVIG as appropriate										
4.9.3	Administer rabies vaccine or IVIG as appropriate										
4.9.4	Immobilize the cervical spine										
4.9.5	Immobilize fractures										
4.9.6	Perform closed reduction of fracture or dislocation										
4.9.7	Administer antibiotics for open fracture										
4.9.8	Perform fasciotomy or escharotomy for compartment syndrome										
4.9.9	Apply three-way dressing for sucking chest wound										
4.9.10	Administer opiate analgesia										
	**Comments:**										

	**Obstetric Interventions**	***Rating*** (1-3)	*Infrastructure*	*Absent Equipment*	*Broken Equipment*	*Sockout (supplies)*	*Training*	*Personnel*	*User fees*	*Opening hours*	*Other (specify in comments)*
4.10.1	Perform assisted vaginal delivery										
4.10.2	Administer uterotonic drug (e.g., oxytocin)										
4.10.3	Perform neonatal resuscitation										
	**Comments:**										

5.0 Essential Resources for Emergency Care: Equipment and Supplies (INSERT HERE)
