# Supplementary material for: Emergency care capacity in Sierra Leone: A multicentre analysis
Source: Afr J Emerg Med. 2024 Feb 6;14(1):58–64. doi: 10.1016/j.afjem.2024.01.003 (PMC10859259; doi:10.1016/j.afjem.2024.01.003)
Supplement: Supplementary file 2 [file mmc2.docx]

**Appendix C.**

List of Key Informants by role; facility units Key Informants cover.

| **Role** | **Unit** |
| --- | --- |
| Anaesthetics Officer | Blood bank |
| Chief Community Health Officer | Clinical Laboratory |
| Community Health Officer | General Female |
| Deputy in Charge | General Male |
| Deputy Matron | HIV unit |
| Facility Monitoring and Evaluation Assistant | Isolation unit |
| Facility Monitoring and Evaluation Manager | Maternity |
| Hospital Secretary | National Emergency Medical Service |
| Laboratory Lead | Outpatients Department |
| Laboratory technician | Paediatrics |
| Matron | Pharmacy |
| Matron Midwife | Special Care Baby Unit |
| Medical Officer | Surgical Female |
| Medical Superintendent | Surgical Male |
| Midwife in Charge | TB Unit |
| Nurse Aide | Theatre |
| Nurse Anaesthetist | Triage |
| Nurse in Charge | Xray |
| Pharmacist |  |
| Pharmacy assistant |  |
| Radiographer |  |
| Referral Coordinator |  |
| State Certified Midwife |  |
| State Enrolled Community Health Nurse |  |
| State Registered Nurse |  |
| Surgical Community Health Officer |  |
| Surgical Officer |  |
| Trainee Nurse |  |
